# Dual RNA-seq analysis provides new insights into interactions between Norway spruce and necrotrophic pathogen *Heterobasidion annosum s.l.*

**DOI:** 10.1186/s12870-018-1602-0

**Published:** 2019-01-03

**Authors:** Andriy Kovalchuk, Zhen Zeng, Rajendra P. Ghimire, Minna Kivimäenpää, Tommaso Raffaello, Mengxia Liu, Mukrimin Mukrimin, Risto Kasanen, Hui Sun, Riitta Julkunen-Tiitto, Jarmo K. Holopainen, Fred O. Asiegbu

**Affiliations:** 1 Department of Forest Sciences, Faculty of Agriculture and Forestry, University of Helsinki, P.O. Box 27, FIN-00014 Helsinki, Finland; 2 Department of Environmental and Biological Sciences, Kuopio Campus, University of Eastern Finland (UEF), P.O. Box 1627, FIN-70211 Kuopio, Finland; 3 Department of Forestry, Universitas Hasanuddin, Jln. Perintis Kemerdekaan Km. 10, Makassar, 90245 Indonesia; 4 Collaborative Innovation Center of Sustainable Forestry in Southern China, College of Forestry, Nanjing Forestry University, Nanjing, China; 5 Department of Environmental and Biological Sciences, Joensuu Campus, University of Eastern Finland (UEF), P.O. Box 111, FIN-80101 Joensuu, Finland

**Keywords:** Dual RNA-seq, Transcriptomics, Norway spruce, *Heterobasidion*, Root and butt rot, Plant defense, Flavonoids

## Abstract

**Background:**

Root and butt rot of conifer trees caused by fungi belonging to the *Heterobasidion annosum* species complex is one of the most economically important fungal diseases in commercial conifer plantations throughout the Northern hemisphere. We investigated the interactions between *Heterobasidion* fungi and their host by conducting dual RNA-seq and chemical analysis on Norway spruce trees naturally infected by *Heterobasidion* spp. We analyzed host and pathogen transcriptome and phenolic and terpenoid contents of the spruce trees.

**Results:**

Presented results emphasize the role of the phenylpropanoid and flavonoid pathways in the chemical defense of Norway spruce trees. Accumulation of lignans was observed in trees displaying symptoms of wood decay. A number of candidate genes with a predicted role in the higher level regulation of spruce defense responses were identified. Our data indicate a possible role of abscisic acid (ABA) signaling in the spruce defense against *Heterobasidion* infection. Fungal transcripts corresponding to genes encoding carbohydrate- and lignin-degrading enzymes, secondary metabolism genes and effector-like genes were expressed during the host colonization.

**Conclusions:**

Our results provide additional insight into defense strategies employed by Norway spruce trees against *Heterobasidion* infection. The potential applications of the identified candidate genes as markers for higher resistance against root and butt rot deserve further evaluation.

**Electronic supplementary material:**

The online version of this article (10.1186/s12870-018-1602-0) contains supplementary material, which is available to authorized users.

## Background

Tree health is a crucial prerequisite of the stable functioning of forest ecosystems. Despite the considerable efforts undertaken to prevent and control the spread of forest trees’ pests and diseases, they continue to pose a serious threat to natural forests and commercial plantations worldwide. Members of the *Heterobasidion annosum* species complex are among the most devastating fungal pathogens in conifer forests of boreal and temperate zones. Growing necrotrophically in the stems and roots of living trees, they greatly reduce the timber quality and make trees more susceptible to windfalls, causing serious economic losses [1]. There is no treatment available for infected trees, and the current control strategies are focused on the post-harvest stump treatment, which prevents the colonization of fresh stumps by *Heterobasidion* fungi. Fungal mycelium can persist in root debris and stumps for decades, and its complete eradication is nearly impossible [1].

Due to its economic importance, the different aspects of interactions between *Heterobasidion* fungi and their host trees have been subjects of numerous studies. The genomes of three species within the species complex were sequenced [2–4], providing valuable resources for comparative genomics studies. At the same time, data from artificial inoculation experiments were used to understand the responses of conifer trees to fungal invasion [5–12]. Results of these experiments demonstrated that jasmonic acid and ethylene signaling pathways play a key role in responses of trees to fungal infection. Induction of components of phenylpropanoid and terpenoid pathways, genes involved in lignin formation and cell wall reinforcement and genes encoding pathogenesis-related (PR) proteins are the principal responses at molecular level. The adaptation of *Heterobasidion* fungi to growth on wood and lignocellulosic substrates also received considerable attention [2, 13–15]. At the same time, much less is known about mechanisms used by *Heterobasidion* fungi to colonize their hosts. One of the limiting factors is the low biomass of pathogen achieved in artificial inoculation experiments, resulting in detection of relatively low number of fungal transcripts [2, 12]. However, study of naturally infected trees at the advanced stages of infection could provide a feasible alternative to the inoculation approach [16].

Despite the good understanding of principal defense responses of conifer trees to *Heterobasidion* infection, much less is known about the factors contributing to their resistance. Traditionally, length of necrotic lesions in tree tissues and the extent of pathogen spread were used to quantify the responses of trees to inoculation [17]. Necrotic lesions alone may not be sufficient as indicator for durable resistance, thus necessitating search for additional markers to complement lesion measurements. The concepts of pathogen exclusion and infection prevention were introduced in the analysis of Lind et al. [18]. However, it is not entirely clear how all these parameters relate to the tree resistance in natural stands. Several candidate genes linked to different aspects of resistance were identified based on analysis of the segregation of SNP markers in F_1_ progeny population [18]. Alternative approach based on genome-wide association analysis of SNP markers with the resistance traits was used by Mukrimin et al. [19]. Further studies showed a possible role of components of flavonoid pathway in the resistance of Norway spruce to *Heterobasidion* infection [20, 21].

The study of host-pathogen interactions greatly benefited from the recent technical advancements. One of the techniques that is particularly suitable for the understanding of molecular mechanisms of interactions between hosts and pathogens is dual RNA sequencing [22, 23]. It allows to detect transcripts with low abundances and is much more sensitive than microarray and Northern blot methods. Furthermore, the method does not require predesigned species-specific probes and allows simultaneous detection of host- and pathogen-specific transcripts in mixed samples. The method has been successfully applied on several plant-pathogen models, illustrating its suitability and clear benefits compared with previously used techniques [24–26].

By applying the dual RNA sequencing approach to study the interactions between Norway spruce and *Heterobasidion* fungi in naturally infected trees, we were able to identify fungal transcripts expressed *in planta*. Among them, we prioritized several candidate genes that might play an important role in the regulation of spruce defense responses [27]. Furthermore, we made a comparison of transcriptional and chemical profiles of asymptomatic and symptomatic spruce trees.

## Results

### Fungal genes expressed during host colonization

**Fig. 1:**
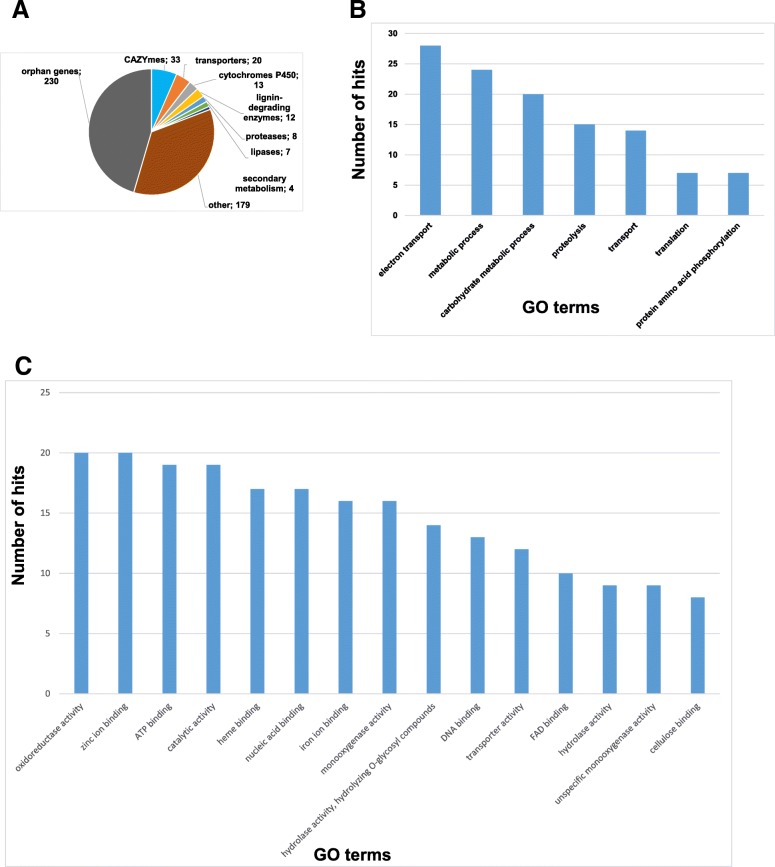
Functional categories of *Heterobasidion* genes expressed during colonization of Norway spruce trees. **a** Pie diagram showing the most abundant functional groups of *Heterobasidion in planta*-expressed genes; numbers of genes assigned to each group are indicated. **b** GO terms in the category “Biological process” with more than 5 hits identified within *Heterobasidion in planta*-expressed genes. **c** Fifteen most abundant GO terms in the category “Molecular function” assigned to *Heterobasidion in planta*-expressed genes

Analysis of RNA-seq data from *Heterobasidion*-infected spruce trees has identified a number of fungal transcripts that were expressed during the colonization of spruce trees (Additional file 1: Table S1). Notably, fungal transcripts were detected both in symptomatic and asymptomatic trees. However, their abundance in the asymptomatic trees was lower than in the symptomatic ones. Functional annotation analysis showed that the large fraction of the identified genes (230 genes) were represented by orphan genes with no information about their biological function (Fig. 1a). Most of the genes falling into this category have no conserved domains. Notably, 49 out of 230 orphan genes were identified as encoding putative effector proteins by EffectorP algorithm [28]. Additionally, three genes from our list were previously selected as putative *Heterobasidion* effectors HaSSP22, HaSSP49 and HaSSP52 [29].

Among other functional groups, a number of genes have a predicted role in the degradation of plant cell wall polymers and lignin decomposition (Fig. 1a; Table 1). We identified 24 genes encoding glycoside hydrolases (GH), 4 genes for carbohydrate esterases (CE) and 3 genes encoding lytic polysaccharide monoxygenases (LPMO). The set of genes with a predicted role in lignin decomposition includes four manganese peroxidases, one laccase, one GMC oxidoreductase, one aryl alcohol oxidase and one cellobiose dehydrogenase.

**Table 1:** List of *Heterobasidion* genes with a predicted role in carbohydrate utilization and lignin degradation, which were upregulated during colonization of Norway spruce trees

Protein ID	Gene name	GenBank accession number	Function	Signal peptide
Carbohydrate-active enzymes (CAZYmes)				
62665		XP_009543268.1	GH2 (candidate β-mannosidase)	yes
107773	gh3.3	XP_009550170.1	GH3 (candidate β-xylosidase)	yes
53076		XP_009551166.1	GH5 (candidate β-glycosidase)	no
181245	gh5.2	XP_009540876.1	GH5 (candidate β-1,4-endoglucanase with N-terminal CBM1 module)	yes
153422		XP_009545732.1	GH5 (candidate β-glycosidase)	yes
62063		XP_009546979.1	GH5 (candidate β-mannanase with N-terminal CBM1 module)	yes
60114	gh6.1	XP_009541827.1	GH6 (candidate cellobiohydrolase with N-terminal CBM1 module)	yes
38802	gh7.1	XP_009544628.1	GH7 (candidate cellobiohydrolase)	yes
426682		XP_009545675.1	GH10 (candidate xylanase)	yes
56987	gh12.4	XP_009553052.1	GH12 (candidate endo-β-1,4-glucanase or xyloglucanase)	yes
65781		XP_009551196.1	GH13 (candidate α-amylase)	no
157474		XP_009550165.1	GH15 (candidate α-1,4-glucanase)	yes
151266		XP_009548433.1	GH20 (candidate exochitinase)	yes
126780		XP_009550237.1	GH27 (candidate α-galactosidase)	yes
12392		XP_009553028.1	GH28 (candidate exopolygalacturonase)	yes^a^
174466		XP_009551306.1	GH28 (candidate rhamnogalacturonan α-L-rhamnopyranohydrolase)	yes^a^
118397		XP_009550976.1	GH28 (candidate polygalacturonase)	yes
125676		XP_009551310.1	GH28 (related to rhamnogalacturonan α-L-rhamnopyranohydrolases)	yes
153387		XP_009550443.1	GH31 (candidate α-glucosidase)	yes
104329	gh45.1	XP_009549290.1	GH45 (distantly related to endo-β-1,4-glucanases)	yes
445737		XP_009549102.1	GH47 (candidate α-mannosidase)	no
37715		XP_009551149.1	GH53 (candidate endo-β-1,4-galactanase)	yes
57301		XP_009553063.1	GH79 (candidate β-glycosidase)	yes
322477		XP_009548553.1	GH115 (candidate xylan α-glucuronidase)	yes
181227	axe1	XP_009552838.1	CE1 (putative acetylxylan esterase)	yes
163550		XP_009545417.1	CE12 (candidate rhamnogalacturonan acetylesterase)	yes
64706		XP_009545882.1	CE15 (candidate glucuronoyl methylesterase)	yes
165050		XP_009553207.1	CE16 carbohydrate esterase	yes
181229	GH61C, GH61.3	XP_009550870.1	LPMO	yes
64994	GH61I, GH61.9	XP_009545898.1	LPMO	yes
105463	GH61A, GH61.1	XP_009550910.1	LPMO	yes
125540		XP_009550793.1	Carbohydrate-binding module family 1 protein with CBM1 domain	yes
322385		XP_009548594.1	endoglucanase E-like protein	no
Lignin-degrading enzymes				
106089	mnp1	XP_009551540.1	manganese peroxidase 1	yes
181068	mnp2	XP_009551541.1	manganese peroxidase 2	yes
101580	mnp5	XP_009547178.1	manganese peroxidase 5	yes
108376	mnp6	XP_009553404.1	manganese peroxidase 6	yes
181063	lcc12	XP_009550378.1	laccase 12 (multicopper oxidase type 1)	yes
163945	aao7	XP_009549981.1	aryl-alcohol oxidase (aao) 7	yes
103798	gor14	XP_009548140.1	GMC family oxidoreductase 14	yes
157537	cdh1	XP_009552848.1	putative cellobiose dehydrogenase 1	yes
426787		XP_009545993.1	related to LigE beta esterase involved in lignin degradation	no
105890	lep1	XP_009551319.1	BNR_4 domain-containing protein specifically expressed on lignin media	yes
105202		XP_009547058.1	similar to *Phanerochaete chrysosporium* 4-O-methyltransferase; may have a role in lignin degradation	yes
446810		XP_009552373.1	similar to *Phanerochaete chrysosporium* 3-O-methyltransferase; may have a role in lignin degradation	no

^a^current protein models are likely truncated at N terminus and do not include a signal peptide sequence, but signal peptide-coding sequence were detected upstream of them

Expression of lignin-degrading peroxidases might be linked to the observed expression of uroporphyrinogen III synthase, an enzyme implicated in heme biosynthesis. Additionally, we identified several genes that might have a role in the detoxification of products of lignin degradation: two genes related to the characterized *Phanerochaete chrysosporium* O-methyltransferases [30], and a gene with a similarity to *Sphingomonas paucimobilis* β-esterase *ligE* [31], as well as *H. irregulare* lignin-expressed protein (lep1) of unknown function.

Other genes that might be relevant for host colonization and substrate utilization by *Heterobasidion* include 7 predicted lipases/carboxyesterases and 8 proteases (6 serine proteases and 2 aspartic proteases). The expressed genes also included 13 genes encoding members of cytochrome P450 family, none of which has been functionally characterized so far. Products of 20 upregulated genes have a putative role in transport, including 14 MFS transporters, 2 amino acid transporters and a single ABC transporter (Fig. 1a). Four of the identified genes might have a role in secondary metabolism: 3 (out of total 13) uncharacterized NRPS-like tridomain enzymes and a terpene cyclase *cyc2*. The last one might be involved in the biosynthesis of the intermediate *en route* to fomajorins and fomannosin toxins [2]. Other genes, which we consider worth mentioning, encode oxalate-producing enzyme oxaloacetase, putative salicylate monooxygenase, a hydrophobic surface-binding protein, hydrophobin, DPBB motif- containing protein that could have a role in binding to plant cell wall, an infection QTL-expressed protein [32] and a LRR domain-containing protein.

Analysis of gene ontology (GO) terms assigned to the identified fungal genes agreed with the conclusions derived from our manual annotation. Among GO terms in the category “Biological processes”, the most abundant ones were “electron transport”, “metabolic process”, “carbohydrate metabolic process”, “proteolysis” and “transport” (Fig. 1b). Similarly, the GO category “Molecular function” terms of “oxidoreductase activity”, “heme binding”, “monooxygenase activity”, “hydrolase activity, hydrolyzing O-glycosyl compounds”, “transport activity” and “cellulose binding” had numerous hits among *in planta*-expressed fungal genes (Fig. 1c). These results emphasize the importance of carbohydrate-degrading enzymes, oxidative enzymes (lignin-degrading peroxidases and cytochromes P450) and transporter proteins during host colonization by *Heterobasidion* fungi.

### Genes showing higher transcript abundance in symptomatic Norway spruce trees

Our analysis of RNA-seq data identified 345 transcripts with significantly higher abundance (FC > 4, FDR < 0.05) in symptomatic trees (Additional file 2: Table S2) and 185 transcripts with higher abundance in asymptomatic trees (see below and Additional file 3: Table S3). Three GO terms (“catalytic activity”, “oxidoreductase activity” and “lyase activity”) were significantly enriched (FDR < 0.05) in the set of genes upregulated in symptomatic trees (Table 2).

**Table 2:** Significantly enriched GO terms in the gene sets with higher transcript abundance in either symptomatic or asymptomatic trees

GO ID	Annotation	*p* value
Asymptomatic trees		
Biological process		
GO:0044699	single-organism process	3∙10^−4^
GO:0051716	cellular response to stimulus	0.011
GO:0007165	signal transduction	0.011
GO:0006855	drug transmembrane transport	0.012
GO:0051234	establishment of localization	0.012
GO:0044765	single-organism transport	0.012
GO:0015893	drug transport	0.014
GO:0008643	carbohydrate transport	0.014
GO:0065007	biological regulation	0.015
GO:0044763	single-organism cellular process	0.018
GO:0006810	transport	0.020
GO:0050896	response to stimulus	0.023
Molecular function		
GO:0022857	transmembrane transporter activity	0.005
GO:0090484	drug transporter activity	0.007
GO:0015297	antiporter activity	0.008
GO:0015238	drug transmembrane transporter activity	0.008
GO:0005215	transporter activity	0.008
GO:0051119	sugar transmembrane transporter activity	0.019
GO:0015291	secondary active transmembrane transporter activity	0.025
GO:0015144	carbohydrate transmembrane transporter activity	0.026
GO:1901476	carbohydrate transporter activity	0.026
Cellular component		
GO:0005887	integral to plasma membrane	3∙10^−4^
GO:0031226	intrinsic to plasma membrane	0.045
Symptomatic trees		
Molecular function		
GO:0003824	catalytic activity	0.002
GO:0016829	lyase activity	0.005
GO:0016491	oxidoreductase activity	0.005

**Fig. 2:**
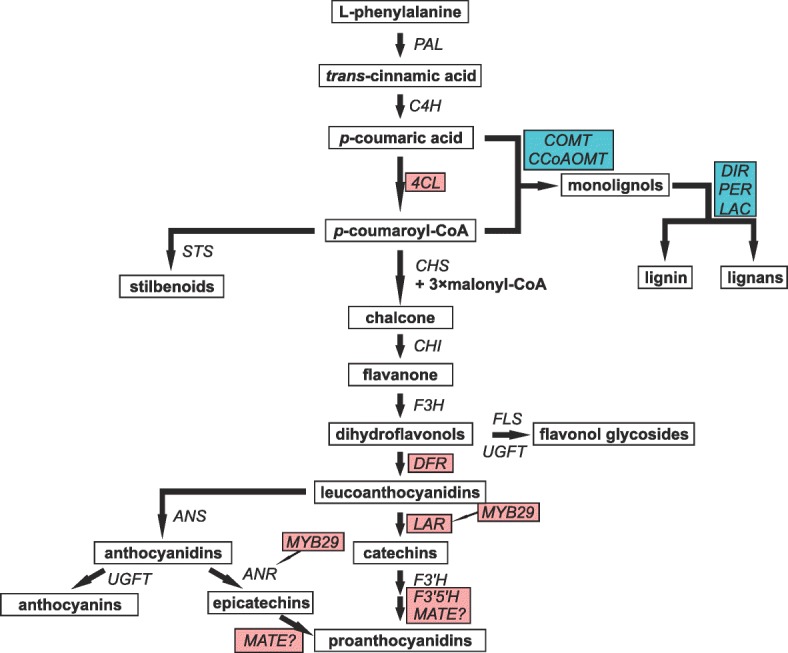
Scheme of the flavonoid biosynthesis pathway with the upstream steps of the phenypropanoid pathway and side branches leading to monolignols and stilbenoids (modified from Mellway et al. [75] and Hammerbacher et al. [44]). Components of the pathway showing higher transcript abundance in asymptomatic trees are boxed in pink, and the ones with higher abundance in symptomatic trees – in turquoise

Functional annotation of the identified genes showed that many genes upregulated in symptomatic trees have a predicted role in cell wall biogenesis and reinforcement, including lignification. This category included 13 secreted peroxidases (also known as class III peroxidases), 4 dirigent-like genes and 1 laccase gene. We also found two putative cellulose synthases, 5 predicted pectin/pectate lyases and a homolog of *A. thaliana* PDR1 ABC transporter, which functions as *p*-coumaryl alcohol exporter and is involved in lignin biosynthesis. Additional genes that might have a role in cell wall biogenesis included a homolog of *A. thaliana* FLA11 protein and three putative members of Trichome birefringence-like protein family. Another important group of upregulated genes was made up by genes encoding predicted receptors (R proteins) and receptor-like kinases (12 and 16 genes, respectively). Four upregulated genes are tentatively involved in phenylpropanoid pathway (Fig. 2): two caffeic acid O-methyltransferases, one caffeoyl-CoA O-methyltransferase and a gene related to *A. thaliana* sinapic acid: UDP-glucose glucosyltransferase, which is required for the establishment of non-host resistance in *Arabidopsis*. Thirteen predicted genes could be assigned to terpenoid biosynthesis pathway: two geranylgeranyl pyrophosphate synthases and 11 genes with similarity to terpene synthases, of which six had the highest similarity to *E*-α-bisabolene synthase, one – to (−)-β-phellandrene synthase, and four – to humulene and longifolene synthases. However, it should be noted that only two of the identified putative terpene synthase genes (MA_1830g0010 and MA_10196363g0010) are full-length gene models. The remaining ones are partial models and some of them could be in fact fragments of the same gene, thus the actual number of upregulated terpene synthases could be somewhat lower.

The genes upregulated in symptomatic trees also included 18 putative transcription factors (6 of which belong to ERF/AP2 family) and 17 pathogenesis-related (PR) proteins (2 PR-1 genes, 5 chitinases, 1 PR-4 gene, 2 PR-10 genes, 1 defensin, 4 genes encoding lipid-transfer proteins, and 2 homologs of *P. sylvestris* Sp-AMP (PR-19)). Several genes could be linked with the JA/ET signaling pathway: a putative homolog of *A. thaliana* cytochrome P450 CYP94B3, which acts as a jasmonoyl-isoleucine-12-hydroxylase and attenuates the jasmonic acid signaling by reducing the levels of JA-Ile, two TIFY domain-containing proteins and a putative ACC oxidase. Other noteworthy genes were two putative members of protein phosphatase 2C family, which is known to have a role in negative regulation of ABA signaling, a homolog of *A. thaliana* trypsin inhibitor KTI1 involved in the modulation of programmed cell death in plant-pathogen interactions, a homolog of *A. thaliana* lipoxygenase LOX1, which confers resistance to *Xanthomonas campestris*, and two homologs of *A. thaliana* PEN3/PDR8 ABC transporter, which has a role in establishment of resistance against fungal pathogens.

### Genes showing higher transcript abundance in asymptomatic Norway spruce trees

The set of genes with higher transcript abundance in asymptomatic trees showed some common features, but also some important differences compared to the genes upregulated in symptomatic trees. GO enrichment analysis demonstrated that several GO terms associated with transmembrane transport, responses to stimuli, signal transduction and biological regulation were significantly enriched (FDR < 0.05) in the set of genes upregulated in asymptomatic trees (Table 2).

Among genes showing higher expression levels in asymptomatic trees, we identified 17 receptor-like kinases and 12 predicted receptor genes. Products of nine genes were assigned to terpenoid biosynthesis pathway: three hydroxymethylglutaryl-CoA reductases and six predicted terpene synthases, of which two showed the highest similarity to (*E,E*)-α-farnesene /(*E*)-β-ocimene synthase, two – to humulene and longifolene synthases, one – to α-terpineol /1,8-cineole synthase and one – to δ-selinene synthase. A number of genes could be linked to phenylpropanoid pathway and its side branches, including three putative caffeic acid/5-hydroxyferulic acid O-methyltransferases, cinnamate-CoA ligase, cinnamoyl-CoA reductase, dihydroflavonol reductase, leucoanthocyanidin reductases *LAR1* and *LAR3* (Fig. 2) and three genes showing similarity to phenylcoumaran benzylic ether reductase. Additionally, three of the identified cytochromes P450 and some of the upregulated UDP-glucosyl transferases might be involved in this pathway. Remarkably, no class III peroxidases, laccases or dirigent-like proteins were upregulated in asymptomatic trees.

Asymptomatic trees also had higher transcript levels of three predicted transcription factors (2 members of ERF/AP2 family and 1 member of MYB family) and three PR-proteins (2 thaumatin-like proteins and 1 germin-like protein). Several identified proteins might have a possible role in the regulation of phytohormone signaling, including one TIFY domain-containing protein, two IAA carboxymethyltransferases, a putative abscisic acid sensor of the PYR/PYL/RCAR family and four MATE efflux family members, which are implicated, among others, in transport of SA and ABA. Other interesting hits that might have a role in spruce defense against pathogens include homologs of *A. thaliana* LAP2 (an aminopeptidase playing key roles in senescence, stress response and amino acid turnover), FMO1 (a protein that promotes resistance and cell death at pathogen infection sites), MLO10 (transmembrane protein involved in cell death and defense responses) and TOXICOS EN LEVADURA 2 (ubiquitin ligase induced after exposure to chitin).

### Chemical profiling of asymptomatic and symptomatic Norway spruce trees

**Fig. 3:**
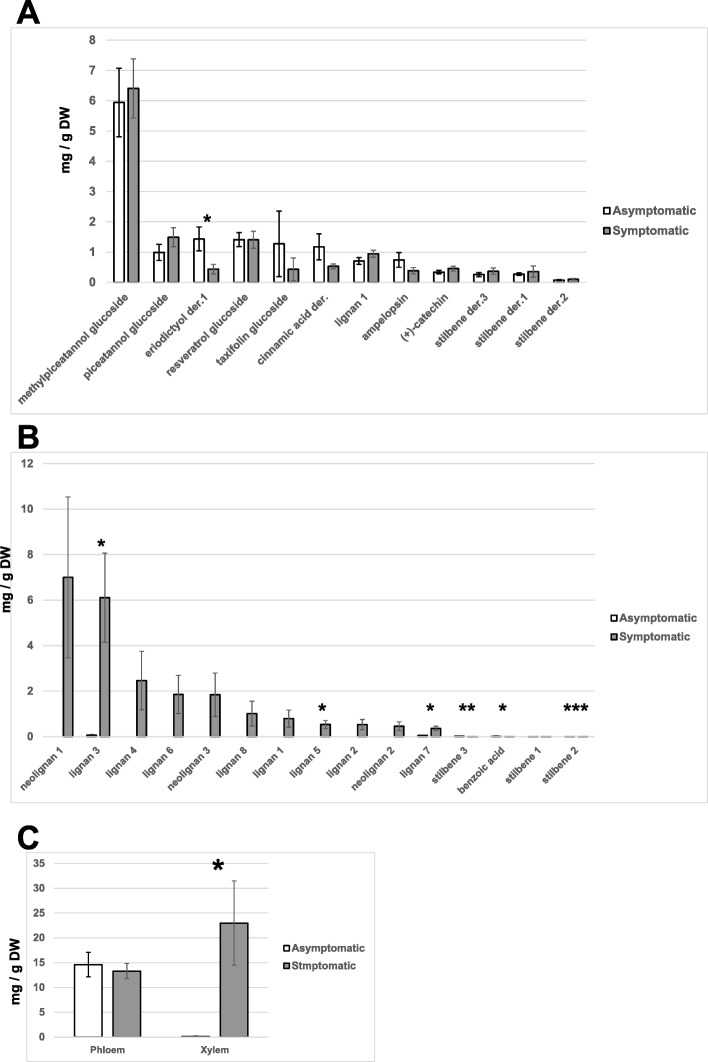
Concentrations of individual phenolic compounds in phloem (**a**) and xylem (**b**) of asymptomatic and symptomatic Norway spruce trees and total concentrations of identified phenolics in the corresponding tissues (**c**). Error bars represent standard errors of mean values. Compounds showing statistically significant differences in their concentrations in asymptomatic and symptomatic trees are marked with asterisks (* *p* < 0.05; ** *p* < 0.01, *** *p* < 0.001)

**Fig. 4:**
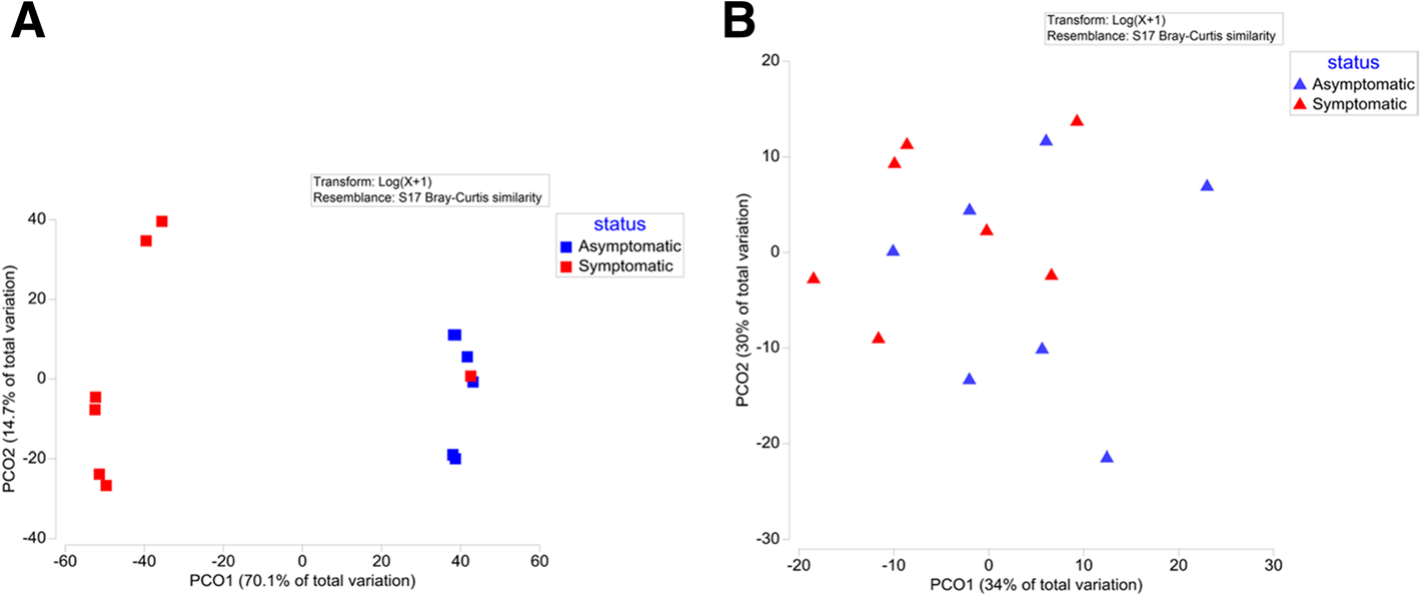
Principle coordinates analysis based on the concentrations of phenolic compounds detected in xylem (**a**) and phloem (**b**) of asymptomatic and symptomatic Norway spruce trees

We performed the analysis of phenolic compounds present in phloem and xylem of asymptomatic and symptomatic Norway spruce trees (Fig. 3). Principal coordinates analysis separated asymptomatic and symptomatic trees based on the profiles of phenolic compounds present in their xylem (Fig. 4a), and PERMANOVA confirmed that this difference was statistically significant (*p* = 0.005). Chemical profiles of phenolic compounds detected in the phloem of asymptomatic and symptomatic trees were not significantly different (*p* = 0.08) (Fig. 4). However, among compounds detected in phloem, the concentration of an uncharacterized derivative of flavanone eriodictyol was about 3.3-fold higher in asymptomatic than in symptomatic trees, and this difference was statistically significant (*p* = 0.038) (Fig. 3a). Total concentration of phenolic compounds in xylem of symptomatic trees was about 160-fold higher than in asymptomatic trees (*p* = 0.031) (Fig. 3c). This pronounced difference was due to the presence of large amounts of lignans and neolignans in symptomatic trees, whereas concentrations of most of them in asymptomatic trees (except for lignans 3 and 7) were below the detection limit (Fig. 3b). At the same time, benzoic acid and stilbenes 2 and 3 were found in xylem of asymptomatic trees (although in low amounts), but could not be detected in all but one symptomatic trees.

**Fig. 5:**
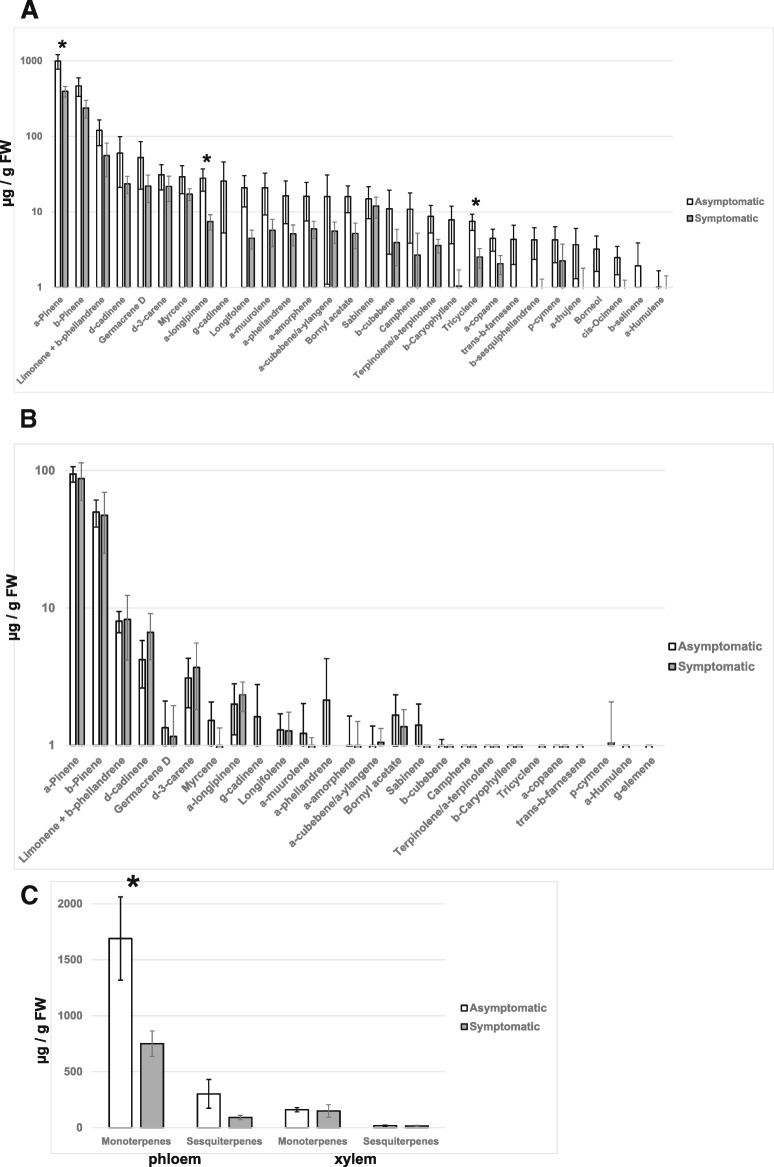
Concentrations of terpenoid compounds in phloem (**a**) and xylem (**b**) of asymptomatic and symptomatic Norway spruce trees and total concentrations of identified mono- and sesquiterpenes in the corresponding tissues (**c**). Error bars represent standard errors of mean values. Compounds showing statistically significant differences (*p* < 0.05) in their concentrations in asymptomatic and symptomatic trees are marked with an asterisk

**Fig. 6:**
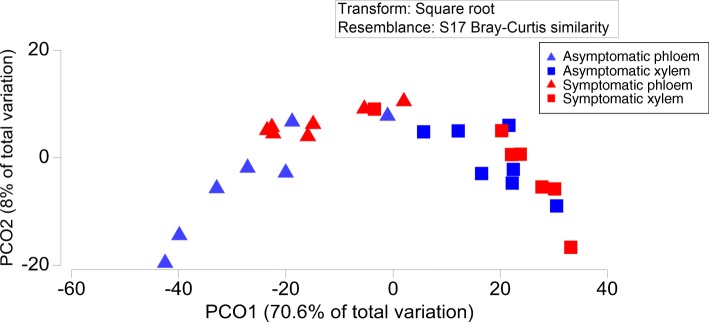
Principle component analysis of terpenoid concentrations in xylem and phloem of asymptomatic and symptomatic Norway spruce trees

We also determined concentrations of 37 terpenoids (17 monoterpenes and 20 sesquiterpenes) in phloem and xylem of asymptomatic and symptomatic Norway spruce trees (Fig. 5). Principal coordinates analysis clearly separated phloem and xylem based on their terpenoid content (Fig. 6). However, PERMANOVA analysis demonstrated that differences among symptomatic and asymptomatic trees were not statistically significant (*p* = 0.064 and *p* = 0.594 for phloem and xylem, respectively). Similar results were obtained when data for monoterpenes and sesquiterpenes were analyzed separately: there were significant differences among xylem and phloem samples, but differences among symptomatic and asymptomatic trees were not statistically significant (*p* > 0.05 in all possible comparisons).

Analysis of data for individual compounds has identified three compounds that were present in higher concentrations in phloem of asymptomatic trees compared with symptomatic trees: α-pinene (*p* = 0.022), tricyclene (*p* = 0.025) and α-longipinene (*p* = 0.048) (Fig. 5a). The total concentration of monoterpenes accumulated in phloem of asymptomatic trees was significantly higher than in symptomatic trees (Fig. 5c). No significant differences among symptomatic and asymptomatic trees were found for xylem concentrations of any of the tested compounds (Fig. 5b).

## Discussion

The interactions between fungi constituting *Heterobasidion annosum* species complex and their natural hosts, various species of conifer trees, have been the focus of numerous studies, owing to the economic importance of these necrotrophic pathogens [1, 2, 5–11, 33]. Recently, these studies were greatly accelerated due to the availability of genome sequences of *Heterobasidion irregulare* [2] and Norway spruce [34]. However, most of the published results were obtained after artificial inoculation of conifer trees with *Heterobasidion*. As *Heterobasidion* fungi cannot penetrate intact surface of conifer stems, artificial wounding is required for the inoculation. The obvious limitation of this approach is that wounding itself causes a strong transcriptional response, which greatly overlaps with tree responses to fungal infection [35].

In this study, we used dual RNA-sequencing approach to investigate the interactions between *Heterobasidion* fungi and Norway spruce trees. Dual RNA-seq is a promising approach to study molecular mechanisms of interactions between plant pathogens and their hosts [24–26]. It was applied to study the interactions between *H. annosum* and Norway spruce previously [12]. However, very few *Heterobasidion*-specific transcripts were detected in that study. This was most likely due to the slow growth of *Heterobasidion* in host tissues, resulting in very low biomass accumulation upon harvesting and low abundance of fungal transcripts in the isolated RNA samples. To overcome the limitations associated with the artificial inoculation of trees with *Heterobasidion*, we decided to focus our analysis at the comparison of naturally infected spruce trees. Similar approach was successfully applied earlier to identify *H. parviporum* genes expressed during colonization of Norway spruce [16]. We recognize that the chosen approach has its own limitations, as we had no information on the duration of the infection process in the selected trees, and we could not fully control environmental variables. Additionally, large size of the sampled tree tissues might have limited the spatial resolution of our experiment, and did not allow us to assess possible differences in transcriptional profiles of *Heterobasidion* hyphae residing within different anatomic regions (i.e., inner and outer heartwood). However, we believe that our experimental design better reflects the situation in managed spruce forests, where trees are constantly exposed to the pathogen. Furthermore, the obtained results indicate that the chosen approach might be valid, as many of the identified fungal and spruce genes could be placed in context of the interactions between the host and the pathogen.

A number of previous studies focused on the adaptation of *Heterobasidion* fungi to growth on lignocellulosic substrates [2, 13–15]. At the same time, the information on *Heterobasidion* genes expressed during growth in living trees is still scarce, mainly due to the methodological difficulties discussed above. Thus, only 11 differentially expressed *Heterobasidion* genes were identified by Lunden et al. [12], 42 upregulated genes were reported by Olson et al. [2], but nearly 400 ESTs from subtraction hybridization libraries were found by Yakovlev et al. [16]. Interestingly, none of the genes reported in a previous study [12] was identified in the present analysis. This could partly be due to the low fungal biomass in that experiment. However, there was some overlap between results of Olson et al. [2] and Yakovlev et al. [16] and the present results. GH5 and GH28 glycoside hydrolases, CE12 carbohydrate esterase, CBM1-containing protein and three MFS transporters were reported as highly expressed in *H. irregulare* during growth in cambial zone of necrotic pine bark [2], and the same genes were detected in the present study. Similarly to the results of Yakovlev et al. [16], we identified manganese peroxidase, laccase, aryl alcohol oxidase and several cytochrome P450 genes, but also numerous proteins of unknown function, which were expressed during colonization of spruce trees. These findings emphasize the importance of carbohydrate- and lignin-degrading enzymes during host colonization by *Heterobasidion*, whereas expressed transporter proteins might be implicated in nutrient acquisition. The observed expression of oxaloacetase is also in agreement with published results [36]. Furthermore, we observed the expression of terpene synthase and NRPS-like genes, which might be involved in the production of toxins by *Heterobasidion* [2]. However, we believe that one of the most interesting findings is the identification of high number of genes of unknown function, expressed by *Heterobasidion* during host colonization. Some of these proteins might be used by the fungus to overcome host defense responses, and, indeed, several of them share a structural organization with known fungal effectors: small secreted cysteine-rich proteins with no conserved domains [29]. The studies on their functions might provide new clues about infection strategies of *Heterobasidion* fungi.

Notably, *Heterobasidion*-specific transcripts were detected both in symptomatic and in asymptomatic trees, although their abundance was lower in the latter. This is in line with the results of our previous metabarcoding study of fungal communities inhabiting the analyzed Norway spruce trees [37]. Our data showed the presence of two OTUs classified as *H. annosum* and *H. parviporum* in the same trees that were used for dual RNA-seq analysis. Both species were detected simultaneously in most of the sampled trees, including the trees selected for the RNA-seq analysis. Identification of *Heterobasidion*-specific transcripts in these trees confirms our previous observations and indicates that fungi were metabolically active in both symptomatic and asymptomatic trees.

Results of our analysis provide further insight into defense mechanisms employed by spruce trees against *Heterobasidion* infection. A high number of predicted receptor (R) genes and receptor-like kinases was upregulated in both groups, and some of them might play an important role in pathogen recognition and in the regulation of defense responses. However, their characterization would require additional experimental efforts. A number of genes involved in lignin formation and cell wall reinforcement (secreted class III peroxidases, dirigent-like proteins and laccase) were induced in symptomatic trees. Induction of these genes is commonly observed in conifers in response to fungal infection and it is activated via JA/ET signaling pathway. Additionally, a set of pathogenesis-related proteins, several components of phenylpropanoid and terpenoid pathways and some further genes with a predicted role in antimicrobial defenses were upregulated in these trees. Nevertheless, induction of all these genes was not sufficient to stop the progression of the infection.

In our opinion, the higher transcript abundance of four genes with high similarity to key regulators of *A. thaliana* cell death and defense responses (LAP2, FMO1, MLO10 and TOXICOS EN LEVADURA 2) in asymptomatic trees is quite remarkable. The identified genes are primary candidates for further studies that would elucidate their role in defense of Norway spruce against *Heterobasidion* fungi and other pathogens.

Results of our experiments provide indications that abscisic acid signaling might be linked to the spruce responses to *Heterobasidion* infection. A gene showing high similarity to *A. thaliana* abscisic acid sensor of the PYR/PYL/RCAR family was upregulated in asymptomatic trees, whereas two putative members of protein phosphatase 2C family, which might be involved in the negative regulation of ABA signaling, had higher expression level in symptomatic trees. To the best of our knowledge, no observations on the possible involvement of ABA signaling in spruce response to *Heterobasidion* infections were made before. In general, the role of ABA in plant pathogen resistance is not completely understood, and both positive and negative effects of ABA on pathogen resistance were reported depending on the studied pathosystem [38]. On one hand, ABA might have a negative effect on plant defense responses against necrotrophic pathogens by suppressing JA/ET-dependent immunity [39]. On the other hand, ABA is often a positive regulator of callose deposition and callose-dependent defense reactions [40–42], and, as such, it contributes to the enhanced pathogen resistance. It would be premature to draw any conclusions from the available data, and further studies will be required to address a possible role of ABA in the resistance of conifer trees against *Heterobasidion* fungi.

We also found higher expression level in asymptomatic trees for two predicted IAA carboxymethyltransferase genes. This enzyme specifically converts IAA to its methyl ester form and in this way lowers the level of IAA. Additionally, four predicted genes encoding transporters of MATE family were upregulated in asymptomatic trees. Members of this family were reported to be involved in the intracellular transport of ABA and IAA, but alternatively they might have their role in flavonoid pathway (see below). We were not able to predict the function of the identified MATE genes based on the available sequence information, and experimental studies will be required to clarify their role.

Our results demonstrated elevated expression level in asymptomatic trees of a number of genes associated with the flavonoid pathway (Fig. 2), which is currently considered as an important component of Norway spruce defense mechanisms. *LAR3* gene encoding leucoanthocyanidin reductase was previously linked with the resistance of Norway spruce to *Heterobasidion* infection [7, 18, 21]. The enzyme encoded by this gene catalyzes the formation of catechins. Notably, the accumulation of catechins in response to *H. annosum* inoculation was reported in Norway spruce genotypes less susceptible to the pathogen [7]. Induction of *LAR* expression was also observed in response to blue-stain fungus *Endoconidiophora polonica* (= *Ceratocystis polonica*) infection [43]. In our experiment, *LAR3* gene and its homolog *LAR1* had significantly higher expression level in asymptomatic trees. Asymptomatic trees also showed higher transcript abundance of a putative dihydroflavonol reductase gene, another enzyme of the flavonoid pathway. Activation of these genes in response to *H. parviporum* inoculation was reported previously [12]. Two of the induced cytochromes P450 showed high similarity to the flavonoid 3′,5′-hydroxylase, which is involved in the conversion of catechin to gallocatechin [44]. Some of the induced MATE transporter-encoding genes could also be associated with flavonoid pathway, as members of this family are implicated in transport of flavonoid precursors of proanthocyanidins [45], but it was not possible to deduce their function from their sequences.

Asymptomatic trees were characterized by higher transcript abundance of MYB family transcription factor *PaMYB29*. MYB proteins make up one of the largest families of transcription factors in plants. They are involved in the regulation of a wide range of developmental programs, metabolic pathways and biotic and abiotic stress responses [46]. Certain MYB proteins are key regulators of phenylpropanoid pathway, and control biosynthesis of monolignols, flavonoids, phenolic acids and stilbenes. MYB-dependent regulation of the phenylpropanoid pathway shows a high degree of complexity, as different MYB factors might have opposite roles and function either as transcriptional activators or as repressors [46]. The role for MYB proteins in the regulation of metabolite fluxes between different downstream branches of phenylpropanoid pathway was proposed [47]. MYB proteins are also involved in the regulation of phenylpropanoid pathway in conifers [48–52]. The available data suggest that this regulation might be achieved via the concerted action of MYB activators and repressors [50]. The role of the transcription factor PaMYB29 in the induction of the genes *PaLAR3* and *PaANR3* in response to biotic stresses was proposed recently [20]. Thus, the higher expression level of *PaMYB29* gene observed in our experiment in asymptomatic trees might be linked to the higher transcript abundance of other genes of the flavonoid pathway.

The observed pattern of gene expression in asymptomatic and symptomatic trees could also reflect the major metabolic differences among those groups. The same precursors derived from phenylpropanoid pathway are used in the biosynthesis of monolignols and flavonoids (Fig. 2) [7]. Higher expression of class III peroxidase, laccase and dirigent genes in symptomatic trees might indicate that these precursors are preferentially used for the formation of lignin and/or lignans, whereas in asymptomatic trees we observe higher expression of components of flavonoid pathway, which, in turn, might indicate increased flow of precursors via this metabolic route. Chemical profiling of phenolic compounds supported this model. The concentration of an uncharacterized derivative of flavanone eriodictyol was significantly higher in asymptomatic trees. At the same time, accumulation of lignans was observed in the xylem of symptomatic trees, whereas concentrations of these compounds in asymptomatic trees remained at the low level. Lignans are a class of phenolic compounds widely distributed among gymnosperms and angiosperms. Chemically, they are dimers of phenylpropanoid units [53], and their biosynthesis starts with the dimerization of two monolignol (most commonly, coniferyl alcohol) units [54]. This reaction is enantioselective, and it is mediated by the oxidative enzymes (laccases and peroxidases) assisted by dirigent (DIR) proteins [55]. Thus, the observed accumulation of lignans in symptomatic trees is in line with the transcriptional activation of genes encoding enzymes required for their biosynthesis, and it agrees with the earlier reports [56]. Lignans might be potential chemical markers for the detection of *Heterobasidion*-infected trees. The regulation of downstream branches of the phenylpropanoid pathway in spruce trees and the contributions of specific MYB transcription factors to it deserve further studies. Taken together, our data support previous observations and highlight some novel details, which could be of importance for further studies on the role of flavonoid pathway in Norway spruce chemical defense.

Another group of metabolites often considered to constitute a part of conifers’ chemical defense are terpenoids. We performed a comprehensive profiling of terpenoid content in xylem and phloem of Norway spruce trees. The observed differences in chemical profiles among asymptomatic and symptomatic trees were not statistically significant, but concentrations of three individual compounds (α-pinene, tricyclene and α-longipinene) were significantly higher in asymptomatic trees. The observed lower terpene concentration in more dynamic phloem could indicate some disturbances of the terpene synthesis by the infection/disease symptoms. At the same time, the lack of similar effects in more stabilized xylem concentrations may suggest that higher susceptibility of the symptomatic trees to the *Heterobasidion* infection was not caused by their inherently lower level of xylem terpenes.

Analysis of RNA-seq data also identified a number of differentially expressed terpene synthase genes. However, it proved difficult to establish unequivocal link between the results of the two analyses, mainly due to the fact that biochemical functions of TPSs cannot be predicted based on sequence similarity [57–59]. A number of Norway spruce TPSs were characterized experimentally [60, 61], however, the only characterized gene among those showing differential expression was (*E*)-α-bisabolene synthase, and this particular compound was not assayed in our chemical analysis. At the same time, no enzymes catalyzing formation of tricyclene and α-longipinene were hitherto reported from Norway spruce, and the gene encoding Norway spruce (−)-α/β-pinene synthase showed no significant differences in transcript abundance. Next to the lack of information on experimentally characterized terpene synthases of Norway spruce, some discrepancy between the results of our chemical and transcriptional analyses could be partially explained by recently reported complex spatial pattern of defense responses in spruce bark [62], which could not be properly resolved using our sampling strategy.

## Conclusions

In summary, the presented data indicate that studies of interactions between fungal pathogens of forest trees and their respective hosts in natural habitats, despite some limitations, can complement the results obtained during artificial inoculations under controlled conditions. In particular, we were able to recover much higher numbers of fungal transcripts compared with the artificial inoculation experiments. Fungal genes implicated in carbohydrate and lignin degradation, transport and secondary metabolism were expressed during the host colonization process. Additionally, about 40% of expressed fungal genes were of unknown function, and some of them resemble fungal effectors and might be used by the pathogen to modulate host defense reactions. Our data support the role of flavonoid pathway in Norway spruce defense against *Heterobasidion*. We also identified several candidate genes related to key regulators of defense reactions in *A. thaliana* and, for the first time, found some indications of the possible role of ABA signaling in defense against *Heterobasidion* infection. These novel insights should contribute to better understanding of the pathosystem of *Heterobasidion* fungi and conifer trees.

## Methods

### Study sites and sample collection

Three Norway spruce (*Picea abies* (L.) Karst.)-dominated forest sites in the municipality of Mäntsälä (Uusimaa region, Southern Finland) were chosen for sampling. The sampling sites and sampling procedures are described in details elsewhere [37, 63]. All three sampling plots are representative examples of managed spruce forest used for commercial timber production and growing at comparable conditions. They are located within an area with a relatively high incidence of *Heterobasidion* infection. Our sampling was performed simultaneously with the tree harvesting. The samples were collected immediately after tree felling. In this way, we could clearly distinguish asymptomatic and symptomatic trees based on presence or absence of wood decay column at stump height (Additional file 4: Figure S1).

In each plot, six spruce trees were selected: three trees showing symptoms of *Heterobasidion*-induced wood decay (further referred to as symptomatic trees), and three trees without decay symptoms (further referred to as asymptomatic trees). Metabarcoding studies of fungal communities associated with the selected trees indicated the presence of two species of *Heterobasidion*, *H. annosum* and *H. parviporum*, in all sampled trees (symptomatic and asymptomatic) [37]. Samples of lower stem (wood disc sectors including bark, sapwood and heartwood) were collected at stump level (Additional file 5: Figure S2). All the samples were kept at − 80 °C until used.

### RNA extraction and sequencing

Out of 18 sampled trees, 10 trees (5 asymptomatic and 5 symptomatic) were randomly selected for RNA-seq analysis. Material from the collected wooden discs was grinded into powder by a sterile liquid nitrogen-cooled IKA A11 basic mill (IKA-Werke GmbH & Co. KG, Germany). Total RNA was extracted from approximately 1 g of grinded tissues (combined sapwood and heartwood) as described elsewhere [64]. For the purpose of RNA isolation, sapwood and heartwood were pooled together, and extraction was performed from the pooled samples. Purified RNA was used for library construction with Illumina TruSeq Stranded Total RNA Library Prep Kit, followed by Illumina paired-end (2 × 75 bp) RNAseq in the facilities of the Institute of Biotechnology (University of Helsinki, Finland). Reads of low quality (Q < 20 in 5-base wide sliding windows) and of adapter contaminations were trimmed by Trimmomatic v.0.35 with minimum length of 38 bp [65]. The quality of trimmed reads was assessed using FASTQC v0.11.2.

The processed reads were mapped against the genome assembly of Norway spruce (v 1.0) [34] (downloaded from ftp://plantgenie.org/Data/ConGenIE/Picea_abies/) and indexed with STAR v.2.5.2b [66]. Raw read count table of the predicted 70,736 gene models was produced by htseq-count script within HTSeq v.0.6.1p1 [67] using the obtained uniquely mapped reads. Furthermore, to identify *in planta* expressed pathogen genes, the processed reads were also mapped against the genome assembly of *Heterobasidion annosum* (v2.0) from Joint Genome Initiative website. The raw reads have been submitted to NCBI SRA under Bioproject PRJNA401308.

### Analysis of RNAseq data

The raw read counts table was loaded into R statistic software [68] and data were analyzed with edgeR package [69]. Read counts with less than 1 read per million in at least 5 of the samples were filtered out [70].

Statistical analysis for differential expression was performed according to the “classic” approach in edgeR for simple experimental design [69, 70]. Briefly, the estimation of the normalizing factor for the libraries and the common/tagwise dispersion were performed by the functions calcNormFactors and estimateCommonDisp/estimateTagwiseDisp, respectively. Finally, the exactTest function was used to calculate genewise exact tests for differences in the means between the two groups of samples generating the list of the differentially expressed features [69]. The cutoff value for FDR was set at 0.05.

Identified differentially expressed Norway spruce genes were manually curated. Annotations were assigned based on the combined results of blastp searches against *Arabidopsis thaliana* protein set, blastp searches against GenBank nr protein sets, limited either to all seed plants or to Pine family (*Pinaceae*) and the presence of conserved domains in the deduced amino acid sequences of the corresponding proteins.

### GO enrichment test

The enriched GO terms in the up-regulated gene lists of asymptomatic and symptomatic trees were retrieved separately by the Enrichment Analysis Tools in ConGenIE.org platform (http://congenie.org/) using all genes in Norway spruce (*Picea abies* v.1.0) as the background. Significantly enriched GO terms in the categories of Biological Process (BP), Molecular Function (MF) and Cellular Component (CC) with at least 3 genes were selected (FDR < 0.05).

### Terpenoid analysis

The analysis of terpenoid compounds from the phloem (inner bark) and the xylem (sapwood + heartwood) of lower stem samples (at stump level) was performed according to the modified method of Kainulainen et al. [71]. Extraction was performed either from 200 mg of phloem samples or 300 mg of xylem samples. Compounds were identified and quantified based on their mass spectra, retention time and authentic standard compounds as described earlier [71].

Concentrations of terpenoid compounds in asymptomatic and symptomatic trees were compared with one-way analysis of variance (ANOVA) in SPSS v24 (IBM, USA). PCoA was used to visualize the structure of host trees terpenoid and transcriptomic profiles. Statistical tests were carried out in PRIMER v.6 [72] with the add-on package of PERMANOVA + [73].

### Phenolic analysis

The analysis of phenolic compounds was performed separately for the phloem (inner bark) and the xylem (sapwood + heartwood) samples taken from lower stem (at stump level). The dried and milled stem material (15 to 20 mg) was used for chemical analyses. The extraction, HPLC quantification and UHPLC-qtof MS identification followed earlier published methods [74]. Concentrations of phenolic compounds in asymptomatic and symptomatic trees were compared with one-way analysis of variance (ANOVA) in SPSS v24 (IBM, USA). PCoA was used to visualize the structure of host trees terpenoid and transcriptomic profiles. Statistical tests were carried out in PRIMER v.6 [72] with the add-on package of PERMANOVA + [73].

## Additional files

Additional file 1: **Table S1.** List of *Heterobasidion* genes expressed during colonization of Norway spruce trees. (XLSX 74 kb)

Additional file 2: **Table S2.** List of Norway spruce genes showing higher transcript abundance in symptomatic trees. (XLSX 39 kb)

Additional file 3: **Table S3.** List of Norway spruce genes showing higher transcript abundance in asymptomatic trees. (XLSX 25 kb)

Additional file 4: **Figure S1.** Representative images of sampled asymptomatic and symptomatic trees showing the extent of *Heterobasidion*-induced wood decay. (TIF 10515 kb)

Additional file 5: **Figure S2.** Scheme illustrating the sampling of Norway spruce material for the transcriptional and chemical analysis. (TIF 698 kb)

## Abbreviations

ABA: Abscisic acid
ABC transporter: ATP-binding cassette transporter
ACC: 1-aminocyclopropane-1-carboxylic acid
AMP: Antimicrobial protein
ANOVA: Analysis of variance
ANR: Anthocyanidin reductase
AP2: Apetala 2
CBM: Carbohydrate-binding module
CE: Carbohydrate esterase
CoA: Coenzyme A
CYP: Cytochrome P450
DIR: Dirigent-like protein
DPBB domain: Double psi beta-barrel domain
ERF: Ethylene-responsive element-binding factor
ET: Ethylene
FC: Fold change
FDR: False discovery rate
FMO: Flavin-dependent monooxygenase
GH: Glycoside hydrolase
GMC oxidoreductase: Glucose-methanol-choline oxidoreductase
GO: Gene ontology
IAA: Indole-3-acetic acid
JA: Jasmonic acid
KTI: Kunitz trypsin inhibitor
LAP: Leucine aminopeptidase
LAR: Leucoanthocyanidin reductase
LOX: Lipoxygenase
LPMO: Lytic polysaccharide monooxygenase
LRR: Leucine-rich repeat
MATE: Multi-antimicrobial extrusion protein
MFS: Major facilitator superfamily
MLO: Mildew resistance locus O
NRPS: Non-ribosomal peptide synthetase
PCoA: Principal coordinates analysis
PDR transporter: Pleiotropic drug resistance transporter
PR proteins: Pathogenesis-related proteins
PYL: PYR1-like protein
PYR protein: Pyrabactin resistance protein
QTL: Quantitative trait locus
R protein: Receptor protein
RCAR: Regulatory component of ABA receptor
RNA-seq: RNA sequencing
SA: Salicylic acid
SNP: Single nucleotide polymorphism
TPS: Terpene synthase
UDP: Uridine diphosphate
