# Supplementary figures and images for: Dual RNA-seq analysis provides new insights into interactions between Norway spruce and necrotrophic pathogen Heterobasidion annosum s.l
Source: BMC Plant Biol. 2019 Jan 3;19:2. doi: 10.1186/s12870-018-1602-0 (PMC6318961; doi:10.1186/s12870-018-1602-0)

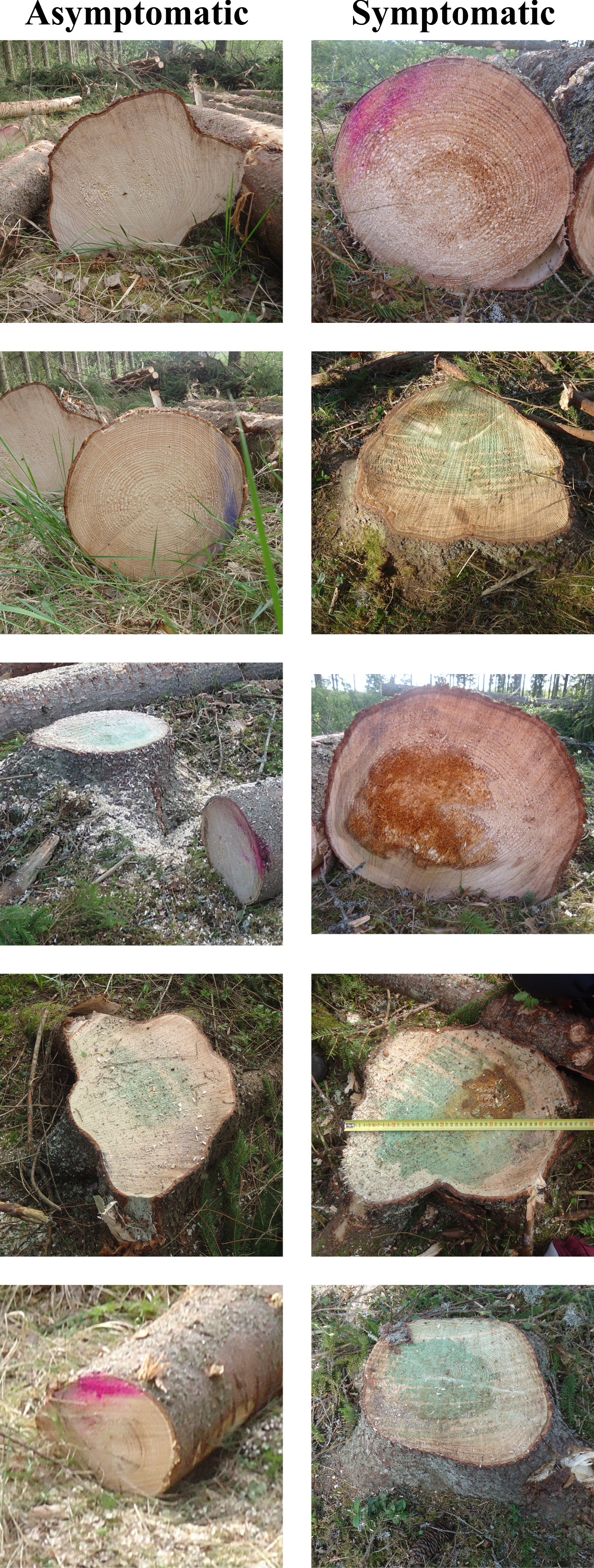

Supplement: Supplementary file 4 — Figure S1. Representative images of sampled asymptomatic and symptomatic trees showing the extent of Heterobasidion-induced wood decay. (TIF 10515 kb) [file 12870_2018_1602_MOESM4_ESM.tif]

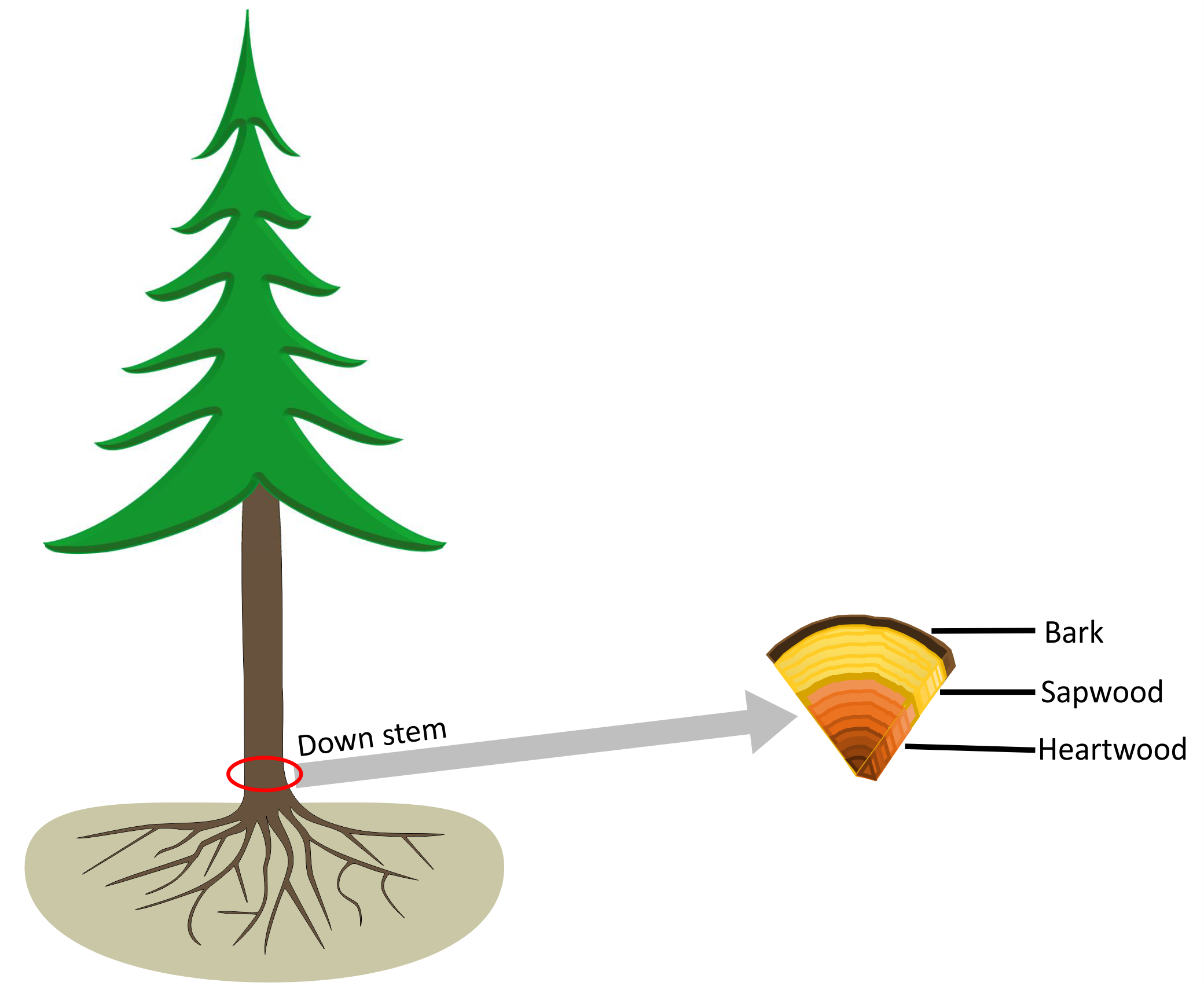

Supplement: Supplementary file 5 — Figure S2. Scheme illustrating the sampling of Norway spruce material for the transcriptional and chemical analysis. (TIF 698 kb) [file 12870_2018_1602_MOESM5_ESM.tif]
